# cGMP Signaling and Vascular Smooth Muscle Cell Plasticity

**DOI:** 10.3390/jcdd5020020

**Published:** 2018-04-19

**Authors:** Moritz Lehners, Hyazinth Dobrowinski, Susanne Feil, Robert Feil

**Affiliations:** Interfaculty Institute of Biochemistry, University of Tübingen, 72076 Tübingen, Germany; moritz.lehners@uni-tuebingen.de (M.L.); hyazinth.dobrowinski@uni-tuebingen.de (H.D.); susanne.feil@uni-tuebingen.de (S.F.)

**Keywords:** cyclic guanosine 3′-5′ monophosphate, nitric oxide, vascular smooth muscle cells, cGMP-dependent protein kinase type I, atherosclerosis, cell plasticity, transdifferentiation, cell fate mapping, imaging

## Abstract

Cyclic GMP regulates multiple cell types and functions of the cardiovascular system. This review summarizes the effects of cGMP on the growth and survival of vascular smooth muscle cells (VSMCs), which display remarkable phenotypic plasticity during the development of vascular diseases, such as atherosclerosis. Recent studies have shown that VSMCs contribute to the development of atherosclerotic plaques by clonal expansion and transdifferentiation to macrophage-like cells. VSMCs express a variety of cGMP generators and effectors, including NO-sensitive guanylyl cyclase (NO-GC) and cGMP-dependent protein kinase type I (cGKI), respectively. According to the traditional view, cGMP inhibits VSMC proliferation, but this concept has been challenged by recent findings supporting a stimulatory effect of the NO-cGMP-cGKI axis on VSMC growth. Here, we summarize the relevant studies with a focus on VSMC growth regulation by the NO-cGMP-cGKI pathway in cultured VSMCs and mouse models of atherosclerosis, restenosis, and angiogenesis. We discuss potential reasons for inconsistent results, such as the use of genetic versus pharmacological approaches and primary versus subcultured cells. We also explore how modern methods for cGMP imaging and cell tracking could help to improve our understanding of cGMP’s role in vascular plasticity. We present a revised model proposing that cGMP promotes phenotypic switching of contractile VSMCs to VSMC-derived plaque cells in atherosclerotic lesions. Regulation of vascular remodeling by cGMP is not only an interesting new therapeutic strategy, but could also result in side effects of clinically used cGMP-elevating drugs.

## 1. Introduction

Cyclic guanosine 3′-5′ monophosphate (cGMP) is a versatile intracellular signaling molecule present in many cell types. It controls numerous physiological processes, from cell contractility, secretion, and permeability to cell differentiation, growth, and survival [1,2]. In mammals, two types of guanylyl cyclases (GCs) have been identified that can generate cGMP from GTP: intracellular NO-sensitive GCs (“soluble” GCs or NO-GCs) [3] and transmembrane GCs (“particulate” GCs or pGCs), such as GC-A, GC-B, and GC-C [4]. The activity of NO-GC is stimulated by the gaseous signaling molecule NO, which is generated by NO synthases (NOSs) [5]. Several pGCs are receptors for peptide hormones. GC-A binds atrial and B-type natriuretic peptide (ANP, BNP), GC-B binds C-type natriuretic peptide (CNP), and GC-C is stimulated by guanylin and uroguanylin [4]. Levels of cGMP are also controlled by cGMP-hydrolyzing phosphodiesterases (PDEs), such as PDE5. The effects of cGMP are mediated by its binding to three classes of cGMP effector proteins: cyclic nucleotide-gated (CNG) cation channels, cGMP-dependent protein kinases (cGKs, also known as PKGs), and cGMP-regulated PDEs [6,7,8]. While CNG channels play a more restricted role in the sensory system, many tissues and cell types express PDEs and cGKs.

Cardiovascular diseases are frequently linked to dysfunctions in vascular smooth muscle cells (VSMCs). VSMCs express various components of the cGMP signaling cascade [3,4,9]. They can generate cGMP in response to NO and natriuretic peptides via NO-GCs and pGCs, respectively, and they express PDEs that degrade cGMP (e.g., PDE5) or cAMP (e.g., PDE3). An interesting aspect is that cAMP hydrolysis by PDE3 is inhibited in the presence of high concentrations of cGMP [7,10]. This inhibition of PDE3 enables crosstalk between cGMP and cAMP signaling in VSMCs in that an increase of cGMP can also result in an elevation of cAMP. A major cGMP effector in VSMCs is cGK type I (cGKI, also known as PKG1), which is encoded by the *prkg1* gene and belongs to the Ser/Thr protein kinase family. cGKI comprises an N-terminal regulatory domain with two cGMP-binding sites and a C-terminal catalytic domain. Two cGKI isoforms are known, cGKIα and cGKIβ, and both are expressed in VSMCs. However, whether cGKIα and cGKIβ have specific functions in vivo is not clear [11,12].

In vascular biology, the classical effects of NO and natriuretic peptides are vasodilation and regulation of blood flow. It is well-accepted that these acute effects are beneficial and mediated by activation of GCs and the cGMP-cGKI pathway in VSMCs [8,9]. NO and natriuretic peptides also have long-term effects on vascular diseases, such as atherosclerosis and restenosis. However, as previously summarized by us [13] and others [14,15], it is still debated whether the cGMP-cGKI axis is involved and, if so, whether it has a positive or negative impact on vascular remodeling and disease. In this review, we will focus on recent in vivo studies that identified a previously unknown form of VSMC plasticity in the context of atherosclerosis and indicate a stimulatory role of the NO-cGMP-cGKI pathway on the growth/survival and phenotypic switching of VSMCs in atherosclerotic plaques. We will also discuss potential reasons for the apparent discrepancy of these studies with a number of studies that reported an anti-proliferative effect of NO, cGMP, or cGKI in VSMCs. Furthermore, we will outline how the use of innovative technologies, such as cGMP imaging and cell tracking, could help to further clarify the role of cGMP in vascular plasticity in the future.

## 2. Role of VSMCs in Physiology and Pathophysiology

### 2.1. Vasodilation via the NO-cGMP-cGKI Pathway

**Figure 1:**
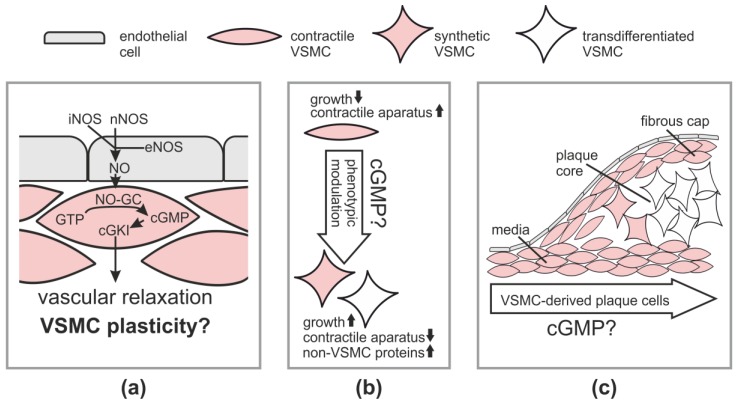
cGMP signaling and vascular smooth muscle cell (VSMC) plasticity. (**a**) The canonical NO-cGMP-cGKI pathway in the vessel wall; (**b**) Concept of VSMC plasticity and (**c**) VSMC-derived cells and VSMC transdifferentiation in atherosclerotic plaques and potential role of cGMP. Note that monocyte-derived macrophages and other plaque cells are not shown. For further explanations, see main text. eNOS, endothelial NO synthase; iNOS, inducible NO synthase; nNOS, neuronal NO synthase.

VSMCs are contractile cells that regulate blood flow and their abnormalities contribute to a range of diseases [16]. Numerous studies have demonstrated that activation of the cGMP-cGKI axis in VSMCs leads to vasodilation [9]. The canonical NO-cGMP-cGKI pathway for vasodilation is depicted in Figure 1a. It comprises generation of NO in the endothelium via endothelial NOS (eNOS) followed by diffusion of NO into VSMCs in the vascular media, where NO-GC is stimulated to generate cGMP. The increased cGMP concentration activates cGKI, which triggers relaxation of VSMCs, likely by phosphorylating several substrate proteins, whose identity has not been completely established [8,11]. Note that vascular NO can also be derived from non-endothelial sources, such as neuronal NOS (nNOS) in neurons or inducible NOS (iNOS) in inflammatory cells [5]. It is generally assumed that activation of the cGMP pathway also dilates resistance-type blood vessels and, thus, lowers blood pressure. Indeed, mouse mutants with impaired NO-GC activity show an elevated basal blood pressure [17,18,19,20]. In contrast, the analysis of cGKI knockout mice indicated that cGKI is not required for basal blood pressure homeostasis, but mediates blood pressure drops in response to the administration of NO-releasing drugs [21,22]. These findings suggest that NO-GC-derived cGMP regulates blood pressure, but the contribution of the cGMP-cGKI downstream pathway to endogenous mechanisms that control blood pressure under basal conditions is probably less important than previously thought.

### 2.2. VSMCs in Vascular Diseases

In addition to blood flow regulation, VSMCs are involved in vascular remodeling during vascular diseases, such as atherosclerosis and restenosis. “Contractile” VSMCs with low proliferative activity and abundant contractile protein expression adapt to the disease situation by changing to highly proliferative “synthetic” cells that have low contractility and produce large amounts of extracellular matrix [23] (Figure 1b). Thus, VSMCs display a remarkable ability to modulate their phenotype in response to changing internal and external stimuli in the context of vascular disease, a process also referred to as phenotypic plasticity. Before discussing the effects of cGMP signaling on vascular plasticity and disease, we will briefly outline the general role of VSMCs in atherosclerosis and restenosis.

#### 2.2.1. Atherosclerosis

Atherosclerosis leads to myocardial infarction and stroke and is the major cause of death in the western world. It is a chronic inflammatory condition that results from complex interactions of modified lipoproteins and various cell types, including monocyte-derived macrophages and cells of the vessel wall [24,25,26]. How each particular cell type contributes to the development of an atherosclerotic lesion is not completely understood [27,28,29,30]. One unsolved issue is the role of mature VSMCs that reside in the vascular media [31,32]. It is well-known that medial VSMCs generate VSMCs that retain contractile protein expression and cover the plaque on its luminal side forming the so-called fibrous cap (Figure 1c). Fibrous cap VSMCs are thought to stabilize the plaque by synthesis of extracellular matrix proteins and, thus, to be beneficial. On the other hand, it was a long-standing matter of debate whether medial VSMCs also contribute to the makeup of the plaque core region, which was assumed to contain mainly monocyte-derived lipid-loaded macrophages, also known as foam cells [33]. Fifteen years ago, we established a Cre/lox-based genetic inducible fate mapping system to track medial VSMCs during atherogenesis in mice. Surprisingly, our initial lineage tracing studies identified a large number of VSMC-derived cells in the core region of atherosclerotic plaques [34,35] and some of them were positively stained for the macrophage marker Mac-2 [34]. These in vivo findings were also consistent with data from cultured VSMCs [36] and led us to put forward the hypothesis that VSMCs can transdifferentiate to macrophage-like cells during atherogenesis [34]. In line with our hypothesis, immunostainings of human plaque sections revealed that some intimal cells co-express markers of smooth muscle cells and macrophages, suggesting the existence of a VSMC–macrophage chimeric cell type in human lesions [37,38]. In 2014, we could indeed demonstrate by definitive lineage tracing and co-staining of VSMC-derived plaque cells with smooth muscle and macrophage markers that medial VSMCs can undergo clonal expansion and convert to macrophage-like cells that have lost classic smooth muscle marker expression and make up a major component of advanced atherosclerotic lesions [39]. This study, as well as the results of other groups that have used similar experimental approaches and reached similar conclusions [40,41,42,43], provide strong in vivo evidence for a major role of VSMC plasticity in atherosclerosis and call for a revised look at the pathogenesis of this devastating disease [33,44]. According to the new model, mature VSMCs that are present in the media of the non-atherosclerotic vessel wall possess the potential to become activated during plaque development and transdifferentiate to macrophage-like cells and perhaps also to other cells that reside within the lesion and have lost expression of VSMC markers (Figure 1c). Interestingly, the VSMC-derived plaque cells have a clonal origin in the vascular media and can make up a major fraction of the intimal cells [39,42,43]. It is likely that previous studies that were based on immunostaining of plaque cells for smooth muscle markers have vastly underestimated the role of VSMC plasticity in atherosclerosis.

#### 2.2.2. Restenosis

Advanced atherosclerotic lesions or plaque rupture can result in occlusion of blood vessels and severe restriction of blood flow [26]. The common approach to restore blood flow in these stenotic vessels is balloon angioplasty or the use of vascular stents. However, these intra-arterial interventions may disrupt normal blood vessel integrity and lead to a long-term risk of restenosis, i.e., the remodeling and eventual re-occlusion of treated arteries. Restenosis is characterized by the formation of a so-called neointima that consists mainly of VSMCs. The exact mechanism of restenosis is still unclear. It involves an inflammatory response and activation of quiescent medial VSMCs with subsequent proliferation and extracellular matrix deposition, thereby forming a neointima [45]. Recent lineage tracing data showed that, similar to atherosclerosis, the neointima is formed by clonal expansion of a small number of medial VSMCs. However, in contrast to the core of atherosclerotic lesions, VSMCs in the neointima exist in a largely contractile and smooth muscle marker-positive state [42]. These findings suggest that neointimal VSMCs may resemble the phenotype of fibrous cap VSMCs in plaques and that the extent of VSMC transdifferentiation is less pronounced in restenosis than in atherosclerosis.

Taken together, vascular remodeling in atherosclerosis and restenosis shows interesting similarities and differences with respect to the behavior of VSMCs. While clonal VSMC expansion is involved in both diseases, VSMC transdifferentiation to other cell types seems to contribute to the formation of atherosclerotic lesions, but not neointima, in the setting of restenosis. Considering the crucial role of VSMC phenotypic plasticity in these vascular diseases, a better understanding of the underlying signaling mechanisms will help to develop novel therapeutic approaches. As discussed in the following sections, shifting the VSMC phenotype from potentially detrimental macrophage-like cells to beneficial fibrous cap cells with drugs that target the NO-cGMP-cGKI signaling pathway in VSMCs could be a novel strategy to treat atherosclerosis.

## 3. cGMP and VSMC Plasticity in Cell Culture

It has long been known that VSMCs grown in vitro lose properties associated with the contractile phenotype (e.g., expression of contractile proteins) and gain “synthetic” features (e.g., proliferation and extensive synthesis of extracellular matrix) [46,47] (Figure 1b). This process is called phenotypic switching or modulation and takes place already during primary culture (e.g., in cells isolated from the aorta, plated on plastic dishes, and grown for 3–7 days) and is further enhanced by subculturing (passaging) of primary VSMCs [48]. Under certain conditions, the loss of contractile marker proteins can be accompanied by expression of marker proteins for other cell types. For instance, after cholesterol loading, cultured VSMCs can modulate to macrophage-like cells [36,49]. Thus, cultured VSMCs appear to provide a useful cell model to study VSMC plasticity, including the process of VSMC-to-macrophage transdifferentiation.

Studies with cultured VSMCs have linked components of the cGMP signaling pathway to VSMC proliferation and marker protein expression, indicating a role of cGMP in phenotypic switching of VSMCs. In particular, the mechanism and therapeutic relevance of cGMP-regulated VSMC plasticity via the NO-cGMP-cGKI axis has been intensively investigated over the past few decades [9,13,15,35,50,51,52]. However, it is still debated if stimulation of this cascade inhibits or promotes the growth of VSMCs. In this section, we will summarize previous data obtained with cultured VSMCs and provide explanations for the seemingly contradicting results.

A general problem in cGMP research is the lack of highly specific activators and inhibitors of pathway components, in particular for use in experiments with intact cells [53]. Many studies with VSMCs applied cGKI activators and inhibitors, but few of them determined the actual efficiency and specificity of the compounds used in the respective experiments. For instance, “cGKI-specific” cGMP analogues may have effects independent of cGKI [54,55]. KT5823, which was frequently used as cGKI-specific inhibitor, might stimulate rather than inhibit cGKI in intact cells [56]; Rp-PET-8-Br-cGMP, which is considered one of the most permeable, selective, and potent cGKI inhibitors, was demonstrated to be a partial agonist of cGKIα rather than an antagonist [57]; and another frequently used cGKI inhibitor, DT-2, was shown to lose its specificity in intact cells [58]. One way to tackle the problems associated with the pharmacological manipulation of cGMP signaling in VSMCs is to combine these tools with genetic deletion or RNA knockdown models of the NO-cGMP-cGKI pathway.

Data obtained with subcultured VSMCs indicated that activation of the NO-cGMP pathway promotes a shift of VSMCs towards the contractile phenotype reflected by reduced proliferation and/or increased contractile marker protein expression [15]. For example, Garg and Hassid [59] showed that NO donors and the membrane-permeable cGMP analogue 8-Br-cGMP (which activates cGKI and other cGMP effectors) reduced the proliferation of subcultured rat aortic smooth muscle cells (RASMCs). Similar results were obtained by application of YC-1 [60], a stimulator of NO-GC [61,62,63]. After adenoviral expression of cGKI in subcultured RASMCs, Chiche et al. confirmed this observation and, in addition, showed an increase in apoptosis after enhancing cGMP signaling [64]. As the overexpression of cGKI in subcultured VSMCs led to increased contractile protein expression (e.g., SM-MHC, calponin), a role for cGKI in maintaining a differentiated/contractile phenotype was postulated [52,65,66].

In contrast to the results obtained with subcultured VSMCs, studies with primary VSMCs revealed a stimulation of VSMC growth and survival by the NO-cGMP-cGKI pathway. Hassid and colleagues demonstrated that the growth-promoting effect of fibroblast growth factor 2 on primary RASMCs was potentiated by NO/cGMP [67]. Interestingly, they did not detect this growth potentiation with cells of higher passages. Another study reported an activation of the mitogen-activated protein kinase pathway (commonly known to be pro-proliferative) by cGMP analogues in freshly isolated RASMCs [68]. We observed a growth-promoting effect of 8-Br-cGMP in primary VSMCs from mouse aorta, and by comparing cGKI-expressing and cGKI-deficient VSMCs, we were able to prove that growth stimulation by cGMP was mediated by cGKI [35]. In the same study, we demonstrated opposing concentration-dependent effects of NO on the growth of primary VSMCs. While a low concentration (0.5 µM) of the NO donor diethylenetriamine NONOate (DETA-NO) enhanced proliferation via a cGKI-dependent pathway, high concentrations of DETA-NO (100 µM) strongly reduced VSMC growth independent of cGKI. A cGMP-independent, anti-proliferative effect of NO in VSMCs was also observed by other groups [50,69]. In sum, there is strong evidence that low/physiological NO concentrations promote VSMC growth via the cGMP-cGKI axis, whereas high/pathophysiological concentrations of NO inhibit VSMC growth in a cGMP/cGKI-independent manner.

Based on the previous studies, it appears that cGMP promotes the growth and survival of primary VSMCs, while it inhibits subcultured VSMCs. Indeed, Weinmeister and colleagues [70] demonstrated that the relatively strong cGKI-dependent growth-promoting effect of 8-Br-cGMP in primary VSMCs (to ≈200–300% of control cells without cGMP) was lost after the first passage and even reversed to a weak growth inhibition in later passages (to ≈90% of control cells without cGMP). These observations imply that passaging of VSMCs results in functional changes of the endogenous NO-cGMP-cGKI signaling pathway and/or more general alterations of the cells’ growth response. Considering the relative magnitudes of cGMP-mediated growth effects, the strong growth stimulation observed in primary VSMCs might be (patho-)physiologically more relevant than the weak inhibition detected in subcultured cells.

Weinmeister et al. [70] also addressed the mechanism underlying growth stimulation of primary VSMCs via the cGMP-cGKI pathway. It was mainly due to a higher efficiency of cell adhesion in the presence of elevated cGMP, while proliferation and apoptosis played minor roles. A known substrate of cGKI that is involved in cell adhesion is the small GTPase RhoA [71,72]. Phosphorylation by cGKI stabilizes RhoA in an inactive cytosolic RhoA-GTP/GDI complex [73,74]. Indeed, the RhoA/Rho kinase signaling pathway was suppressed by activation of the cGMP-cGKI pathway in VSMCs. This led to activation of β_1_/β_3_ integrins and enhanced adhesion of primary VSMCs [70].

Recently, Segura-Puimedon and colleagues [50] analyzed the phenotype of VSMCs from knockout mice lacking the α1 subunit of the cGMP-generating NO-GC. Compared to wild-type VSMCs, NO-GC knockout cells showed less proliferation and migration and increased expression of contractile marker proteins. These data are in line with the results obtained with cGKI knockout VSMCs [35]. Together, these in vitro studies support a model in which increased NO-cGMP-cGKI signaling promotes the growth and survival of VSMCs and stimulates their modulation towards a synthetic phenotype (Figure 1b).

## 4. cGMP and Vascular Diseases

The critical role of NO and natriuretic peptides in the cardiovascular system has been known for a long time. NO-releasing organic nitrates have been used for the treatment of angina pectoris for more than a century. Novel functions of cGMP and clinical applications of cGMP-based drugs are continuously being discovered [75,76]. Indeed, drugs that increase cGMP concentration have emerged as one of the most successful areas in recent drug development and clinical pharmacology [77,78]. For example, the PDE5 inhibitor sildenafil is used for erectile dysfunction, the GC-C agonist linaclotide for chronic idiopathic constipation and irritable bowel syndrome, and recently a combination drug consisting of valsartan (an angiotensin II receptor blocker) and sacubitril, which inhibits the ANP-/BNP-degrading endopeptidase neprilysin, was approved for use in heart failure [79]. The NO-GC stimulator riociguat is used for several forms of pulmonary hypertension [80]. Currently, a plethora of preclinical and clinical studies are testing NO-GC stimulators and activators for their therapeutic potential in various diseases, including heart failure, aortic valve calcification, achalasia, and fibrosis [75,78,80].

The clinical data is supported by genetic association studies, which have implicated dysfunctions of the cGMP signaling cascade in hypertension, coronary artery disease, and myocardial infarction in humans [81]. Importantly, even subtle changes due to genetic variants in components of this pathway (e.g., NO-GC, ANP, BNP; PDEs) significantly influence blood pressure and cardiovascular disease risk [82,83,84,85,86]. Interestingly, genetic mutations in NO-GC or cGKI found in humans are causally associated with altered vascular structure and remodeling [87,88]. However, the effects of genetic polymorphisms on the expression level and/or enzymatic activity of the respective proteins are not always known and, because the genetic alterations are present in all cells of the body, it is difficult to identify the causative cell type(s) (e.g., endothelial cells, platelets, VSMCs, etc.). Although it is commonly thought that the net effect of cGMP signaling on cardiovascular disease is protective, it is conceivable that an increase of cGMP in different cell types can have different, and even opposing, effects on disease progression. In particular, the in vivo relevance of vascular plasticity regulated by the NO-cGMP-cGKI cascade is not completely understood.

In recent years, knockout mouse models for NOS, NO-GC, and cGKI have been established. Table 1 summarizes the vascular phenotypes of the respective mouse mutants. Conventional null mutants that lack the β1 subunit of NO-GC [89] or cGKI [22] “chronically” in all cells have a strongly reduced life span limiting their use for long-term experiments, such as the analysis of atherosclerosis. Another drawback of conventional knockout technology is the fact that the gene mutation is present in all cells, making it difficult to assign a phenotype to a specific cell type. To circumvent problems associated with global gene knockouts, time- and tissue-specific mutagenesis utilizing the Cre/lox recombination system can be performed [90], and conditional alleles of NO-GC [89] and cGKI [91] have been generated.

### 4.1. Role of cGMP in Atherosclerosis

Several genetic mouse studies investigated the role of NO-cGMP signaling for the development of atherosclerotic plaques, a process strongly dependent on VSMC plasticity. Interestingly, the phenotypes of NOS knockout mice indicated an ambivalent role of NO in atherosclerosis (Table 1). While NO generated by eNOS [92,94] and nNOS [101] was atheroprotective, NO synthesized by iNOS, which is upregulated under inflammatory conditions and generates high amounts of NO, promoted atherosclerosis [103,104]. Interestingly, publications on the effects of pharmacological activation of NO-GC on atherosclerosis are scarce [62]. One study reported that the NO-GC stimulator YC-1 prevents foam cell formation and atherosclerosis [111]. The opposing effects of NO might be related to the different spatiotemporal profile (cell types, time) and quantity of NO generation by eNOS/nNOS versus iNOS [112]. These results are also consistent with in vitro data showing that NO can promote and inhibit VSMC growth via cGMP-dependent and cGMP-independent pathways, respectively (see Section 3, “cGMP and VSMC Plasticity in Cell Culture”). Clearly, the interpretation of in vivo knockout phenotypes is complicated if the gene mutation is present in all cells and/or the mutant mice display multiple phenotypes that could influence each other. For instance, eNOS-deficient mice are also hypertensive and it is debated whether or not this may be another reason for their enhanced atherosclerosis independent of a potential direct effect of NO on VSMC plasticity [92,93].

Using an inducible smooth muscle-specific cGKI knockout model that was combined with genetic tracking of VSMC fate during plaque development, we demonstrated that the cGMP-cGKI axis in VSMCs promotes the growth of atherosclerotic lesions [35]. After postnatal ablation of cGKI in VSMCs, mutant mice developed smaller plaques. In these plaques, cells derived from cGKI-deficient VSMCs were almost exclusively located in the media but not the plaque core region. In contrast, cells derived from wild-type VSMCs were found in both the vascular media and inside the plaque. These cell-fate mapping data indicated that cGKI is involved in the development of VSMC-derived plaque cells, which were later identified as macrophage-like cells (see Section 2, “Role of VSMCs in Physiology and Pathophysiology”). Thus, it is tempting to speculate that cGMP-cGKI signaling promotes smooth muscle-to-macrophage transdifferentiation in atherosclerosis (Figure 1c).

In line with a pro-atherogenic role of the NO-cGMP-cGKI signaling cascade, Segura-Puimedon and colleagues recently demonstrated that deletion of the α1 subunit of NO-GC led to a reduced size of atherosclerotic plaques [50]. Their in vitro and in vivo results clearly confirm and further extend the atherosclerosis data obtained with cGKI mouse mutants [35]. Together, these studies suggest that the activated NO-cGMP-cGKI pathway promotes the phenotypic switching of VSMCs from a contractile/quiescent phenotype to a synthetic/proliferative state including macrophage-like plaque cells. It is important to note that cGMP-dependent plaque growth is not necessarily an unfavorable process. Indeed, the cGMP-mediated increase in lesion size is associated with an altered plaque composition, which may or may not stabilize the plaque. These questions as well as the role of NO-GC and cGKI in multiple cell types involved in atherosclerosis (e.g., VSMCs, endothelial cells, platelets, immune cells) should be further addressed in future studies with conditional knockout mice.

### 4.2. Role of cGMP in Restenosis

Paralleling the effects of NOS-derived NO in atherosclerosis, studies using knockout models in the setting of restenosis (Table 1) demonstrated vasculoprotective effects of eNOS [95,96,97] and nNOS [102] and a vasculoproliferative effect of iNOS [105]. The dual role of NO in vascular remodeling might be the reason why NO-releasing drugs, to our knowledge, have not been reported to exert beneficial effects on atherosclerosis or restenosis. In contrast to the strong in vivo evidence supporting a role of the NO-cGMP-cGKI axis in atherosclerosis, data proving an influence of this signaling pathway on the development of neointima during restenosis are scarce. Adenoviral transduction of the constitutively active kinase domain of cGKI led to an attenuated formation of neointima in vascular injury models of rat and swine [113]. It is important to note that this kinase construct is not regulated by cGMP and lacks the N-terminus, which is responsible for substrate specificity [8,9]. In the same study, the transduction of the full-length cGKIβ isoform did not affect the formation of neointima. Lukowski and colleagues showed that smooth muscle-specific ablation of cGKI and treatment of wild-type mice with the PDE5 inhibitor sildenafil had no significant effect on restenosis in various models of vascular injury, except for a slight reduction of neointima formation in a short vessel segment of cGKI mutants after wire injury of the carotid artery [51].

Pharmacological approaches applying the NO-GC stimulator YC-1 [114,115] or the NO-GC activator cinaciguat [116] indicated a vasculoprotective role of NO-GC activation in rat models of restenosis. It is, however, not clear whether these effects were related to drug action on VSMCs and/or other cell types. It should be considered that pharmacological activation of NO-GC can exert hypotensive [61,62,117] and anti-inflammatory effects [111,118], which could influence restenosis. Furthermore, YC-1 is known to have cGMP-independent effects [62,117]. In contrast to the pharmacological studies, Vermeersch et al. reported that NO-GC might promote restenosis based on their finding that global knockout of the NO-GC α1 subunit resulted in a reduction of neointima formation in male mice [107]. Together with the analysis of cGKI mouse mutants, these genetic knockout studies suggest that the NO-cGMP-cGKI cascade is not critically involved in the regulation of VSMC growth during restenosis. If at all, this pathway may slightly promote rather than inhibit neointima formation, which points to the same direction as the growth-promoting effect of cGMP during atherosclerosis. However, the effects of cGMP on restenosis appear relatively weak and it is unlikely that the effects of NO on restenosis are mediated by the cGMP-cGKI pathway. Presumably, the spatiotemporal profile, the amount of NO synthesized, and the source of its production after vascular injury result in the activation of alternative mechanisms, such as redox regulation of target proteins [119]. The preclinical evidence summarized above also indicates that the contribution of cGKI-mediated mechanisms to vascular remodeling is context-specific, and arguably more important in atherosclerosis than in restenosis. A disease-specific role of the cGMP-cGKI axis is plausible considering our hypothesis that this pathway acts on smooth muscle-to-macrophage transdifferentiation (see above, this section), a process that seems to contribute to the formation of atherosclerotic lesions, but not neointima in the setting of restenosis (see Section 2, “Role of VSMCs in Physiology and Pathophysiology”).

### 4.3. Role of cGMP in Angiogenesis

Another process influenced by the phenotypic plasticity of VSMCs is the formation of blood vessels. It is known that NO induces postnatal neovascularization through both angiogenesis (the development of new blood vessels derived from existing vessels) and vasculogenesis (blood vessel formation de novo from progenitor cells), but the respective mechanisms are still unclear [112]. Hindlimb ischemia is a common model to investigate blood vessel formation during pathological conditions, as angiogenesis is the natural response to restore blood supply after ischemia. In this model, the pro-angiogenic effect of NO was demonstrated by using a NOS inhibitor [120] or eNOS knockout models with impaired NO generation [100] (Table 1). Pro-angiogenic effects of eNOS [98,99] and iNOS [106] were also observed in other models of neovascularization. A recent study identified NO-GC as a component of cGMP-mediated angiogenesis [108]. By comparison of ischemia-induced angiogenesis in global, endothelial-, and smooth muscle-specific NO-GC β1 knockout mice, the authors showed that NO-GC expression in VSMCs, but not endothelial cells, improved neovascularization. In line with the role of NO and NO-GC, several studies also suggested a pro-angiogenic function for cGKI. Yamahara and colleagues reported that angiogenesis in response to hindlimb ischemia was increased in mice that overexpressed cGKIα, while it was significantly reduced in heterozygous cGKI knockout mice [110]. Aicher et al. confirmed the pro-angiogenic cGKI effects observed by Yamahara using a cGKIα leucine zipper mutant unable to interact with downstream targets [109]. Furthermore, this study reported reduced blood vessel formation of cGKI-null mutants in a disc neovascularization model. Moreover, Senthilkumar and colleagues proposed a cGKI-dependent pro-angiogenic effect of sildenafil [121]. Taken together, these studies strongly suggest a pro-angiogenic function of the NO-cGMP-cGKI pathway.

## 5. Limitations and Future Directions

Clearly, the present data call for more studies on the mechanisms and therapeutic relevance of cGMP-regulated vascular remodeling. These studies should address how cGMP signaling affects VSMC phenotype and vice versa. The relevance of cGMP-triggered VSMC transdifferentiation should be analyzed in detail as well as the effects of cGMP on other cell types involved in vascular disease, such as endothelial cells, platelets, and immune cells. To answer these and other interesting questions, it will be instrumental to monitor the spatiotemporal profile of cGMP signals generated in living vascular cells and tissues, and to track the cells’ fate after endogenous or pharmacological modulation of the cGMP pathway.

A major obstacle in drawing a complete picture of cGMP signaling in VSMCs is their phenotypic heterogeneity. It is likely that the initial state of a VSMC influences the effect of cGMP on it. Therefore, it is important to investigate cGMP signaling at the single-cell level. To some extent, this is possible by classical immunostainings of VSMC populations in cell culture or tissue sections for cGMP, “marker” proteins, or other proteins of interest. However, this method does not allow one to follow dynamic changes and it is limited by the availability of specific antibodies. With the development of genetically-encoded fluorescent cGMP sensors, it is now possible to visualize cGMP in living cells by fluorescence microscopy [122,123]. Recently, we have generated transgenic mice expressing the cGMP indicator cGi500 [124] either ubiquitously or in specific cell types [125]. The cGMP sensor mice are a convenient source to isolate sensor-expressing primary cells [122,125] and tissues [126,127] for ex vivo cGMP imaging, or they can be used directly for intravital cGMP imaging in vivo [128]. Thus, it is possible to monitor dynamic cGMP changes in real time in individual VSMCs in culture or in a living tissue/animal under close-to-native conditions. In contrast to conventional end point cGMP assays, which measure cGMP in cell/tissue extracts, the cGMP sensor mice allow for monitoring of cGMP levels in single cells in response to multiple stimulations [122,125]. This will help to correlate cGMP responses elicited by different cGMP-elevating agents with the phenotypic state of individual VSMCs and, thereby, deepen our understanding of the role of cGMP in phenotypic plasticity. Furthermore, the cGMP-modulating effects of PDE inhibitors or other substances can be investigated at the single-cell level as was recently shown for neurons [129]. Besides this, spatiotemporal differences of cGMP signals can be analyzed in intact tissues, which has already provided new insights about cGMP signaling in the cochlea [126] and oocytes [127].

It is increasingly recognized that previous studies have underestimated the importance of VSMC phenotypic plasticity in atherosclerosis and the impact a single VSMC can have on disease progression. With the use of genetic cell-fate mapping, it was shown that individual medial VSMCs can expand clonally and transdifferentiate to macrophage-like cells in atherosclerotic plaques. These studies also showed that classification of cell types exclusively via immunostainings for “specific” marker proteins, which they might lose or gain during phenotypic switching, can lead to misinterpretations concerning the cells’ origin. We anticipate that genetic lineage tracing will continue to discover new aspects of VSMC plasticity in vascular diseases, such as conversion to bone-like cells and other cells. Recently, a new mouse model for cell tracking with positron emission tomography (PET) was described [130]. This cell tracking mouse allows for non-invasive imaging of specific cell types by PET and should further improve data quality with a reduction in the number of animals needed. Importantly, we can now track VSMCs over time in an individual living animal without the need to sacrifice it. In the future, combining cGMP imaging and VSMC tracking will help to improve our understanding of cGMP’s role in VSMC plasticity in vivo.

## 6. Conclusions

The cGMP signaling pathway has a strong impact on human cardiovascular physiology and pathophysiology and is an attractive drug target to tackle an array of major human diseases. As discussed in this review article, in vitro and in vivo data support an important role of the NO-cGMP-cGKI axis in vascular remodeling, particularly in the context of atherosclerosis and angiogenesis. It is likely that many of these effects are related to stimulation of VSMC growth and plasticity by this signaling pathway (Figure 1b). A major future challenge is to evaluate the relevance of cGMP-regulated vascular plasticity for human health and disease. Preclinical studies strongly support the notion that the cGMP-cGKI axis increases VSMC growth and survival. Similar growth/survival-promoting effects of cGMP have been reported in other cell types, including cardiomyocytes [131,132,133,134], hematopoietic [135] and vascular [109] progenitor cells, stroma cells in the bone marrow [136], erythrocytes [137], platelets [138], osteoblasts [139], sensory hair cells [140], and melanoma cells [141]. Thus, we propose that stimulation of cell growth and survival by cGMP is a common mechanism in many cell types and tissues. The growth-promoting effects of cGMP could be highly relevant for pharmacotherapies. Inhibition of the cGMP-cGKI pathway could be a novel strategy to treat proliferative diseases, such as atherosclerosis and cancer. Stimulation of this pathway might counteract cell degeneration and death in settings such as heart attack, stroke, or noise-induced hearing loss. However, cGMP-elevating drugs used in clinics might also have unwanted side effects related to stimulation of cell growth and survival. Indeed, two recent clinical studies reported that use of PDE5 inhibitors in men is linked to a modest increase in melanoma risk [142,143]. We [141] and others [144] discovered a growth-promoting cGMP pathway in melanoma cells that might provide a mechanistic basis for this clinical finding [145]. In the future, it will be interesting to evaluate the effects of cGMP-modulating drugs on atherosclerosis and other diseases that are associated with cell growth and plasticity.

## Figures and Tables

**Table 1:** Genetic mouse models of NO-cGMP-cGKI signaling and their vascular phenotypes.

Gene	Mouse Model	Effect of Mutation on Vascular Remodeling	References
eNOS	Null mutation	Enhanced atherosclerosis on ApoE^−/− 1^ background	[92,93,94]
		Enhanced neointima formation after vascular injury	[95,96,97]
		Impaired angiogenesis	[98,99,100]
nNOS	Null mutation	Enhanced atherosclerosis on ApoE^−/−^ background	[101]
		Enhanced neointima formation after vascular injury	[102]
iNOS	Null mutation	Reduced atherosclerosis on ApoE^−/−^ background	[103,104]
		Reduced neointima formation after vascular injury	[105]
		Reduced pathological neovascularization in the ischemic retina	[106]
NO-GCα1-subunit	Null mutation	Reduced atherosclerosis on ApoE^−/−^ background	[50]
		Reduced neointima formation after vascular injury in male mice	[107]
NO-GCβ1-subunit	Smooth muscle-specific knockout (tamoxifen-inducible)	Reduced arteriogenesis in hindlimb ischemia model	[108]
	Null mutation	Reduced arteriogenesis in hindlimb ischemia model	[108]
cGKI	Smooth muscle-specific knockout (tamoxifen-inducible)	Reduced atherosclerosis on ApoE^−/−^ background	[35]
	Smooth muscle-specific knockout	No effect on neointima formation after vascular injury	[51]
	Null mutation	Reduced angiogenesis	[109,110]

^1^ Apolipoprotein E (ApoE).
